# Hashimoto’s thyroiditis both promotes and protects against papillary thyroid cancer

**DOI:** 10.3389/fendo.2026.1898677

**Published:** 2026-09-02

**Authors:** Xin Lin, Zhi Chen, Dandan Song, Jie Shen

**Affiliations:** 1 Department of Endocrinology and Metabolism, The Third Affiliated Hospital of Southern Medical University, Guangzhou, Guangdong, China; 2 Department of Endocrinology and Metabolism, The Eighth Affiliated Hospital of Southern Medical University (The First People’s Hospital of Shunde), Foshan, Guangdong, China; 3 GuangDong Engineering Technology Research Center of Metabolic Disorders Interdisciplinary Precision Prevention and Digital Healthcare, The Eighth Affiliated Hospital of Southern Medical University (The First People’s Hospital of Shunde), Foshan, Guangdong, China

**Keywords:** Hashimoto’s thyroiditis, papillary thyroid cancer, autoimmune thyroid disease, immune microenvironment, CD4-positive T cells, trace element, tumor immunology

## Abstract

The growing body of evidence suggests a complicated association between papillary thyroid cancer (PTC) and Hashimoto’s thyroiditis (HT). Although HT is a major risk factor for PTC development, it is also associated with improved patient outcomes. However, the molecular mechanisms between these two diseases remain unclear. This narrative review summarizes clinical evidence that connects HT with PTC and examines how these two conditions interact. It explores the involvement of CD4^+^ T-cell subsets, regulatory T cells, CD8^+^ T cells, tumor-associated macrophages, and key immunological molecules in remodeling the tumor immune microenvironment (TIME) during HT. We also discuss the contributions of genetic predisposition, environmental factors, thyroid-stimulating hormone and autoantibodies, non-coding RNA regulatory networks, and the gut microbiota to the relationship between the two diseases. Further research is needed to fill the gaps in our understanding of HT and PTC linkages, aiming to provide a theoretical foundation for developing personalized therapies that target the TIME.

## 1 Introduction

Among all autoimmune diseases targeting the thyroid gland, Hashimoto’s thyroiditis (HT), sometimes referred to as chronic lymphocytic thyroiditis (CLT), is the most common, affecting approximately 7.5% of the global population and posing a substantial public health burden (1, 2). Clinical diagnosis of HT relies on the presence of thyroid-specific autoantibodies and characteristic hypoechogenicity on thyroid ultrasound, whereas current management remains centered on levothyroxine replacement due to the absence of directed intervention for the underlying autoimmune process (1). Concurrently, accounting for 90% of thyroid cancers (3), papillary thyroid cancer (PTC) has also emerged as a condition of growing public and clinical concern, driven by its rapidly rising incidence and increased diagnostic scrutiny. Given its indolent biological behavior and excellent long-term prognosis — with a 93% 10-year disease-specific survival rate — non-surgical management strategies, such as thermal ablation and active surveillance, have become significant alternatives for PTC (4).

Since Dailey and colleagues first described the coexistence of HT and PTC in 1955, accumulating clinical and mechanistic evidence has strongly supported a non-random, pathophysiologically linked relationship between these two conditions (5). HT appears to play a dual role in PTC. While epidemiological evidence consistently links HT to a significantly increased risk of PTC development, when both disorders coexist, PTC tends to display less aggressive features, including reduced lymph node metastasis and more favorable long-term outcomes (6). This paradoxical dual effect has become a central theme in recent research. Current understanding suggests that this association may involve multiple factors, including the unique tumor immune microenvironment (TIME), genetic predisposition, environmental exposures, and epigenetic regulation (7). In this narrative review, we aim to synthesize the available evidence and provide a comprehensive analysis of the underlying immunological and molecular mechanisms.

## 2 Clinicopathological correlations between Hashimoto’s thyroiditis and papillary thyroid cancer

### 2.1 Hashimoto’s thyroiditis as a risk factor for papillary thyroid cancer

While HT confers an elevated risk of several cancer types, its association with PTC is the most clearly established, with no comparable link having been observed for follicular, medullary, or anaplastic thyroid cancer (8). PTC arising in the context of HT (hereinafter referred to as HT-PTC) exhibits higher rates of multifocality and bilateral distribution (6, 9), and nearly half of multifocal thyroid cancers of independent clonal origin are accompanied by HT, implicating the HT-shaped immune milieu as a facilitator of multiclonal tumor development (10).

The coexistence of HT and PTC is particularly prominent among children and adolescents. In this population, the prevalence of concurrent autoimmune thyroiditis (AIT) reaches 48.9% and increases with age. AIT is clinically equivalent to HT, as defined in that study. Furthermore, AIT is strongly associated with the diffuse sclerosing variant of PTC in children (OR = 4.74) (11), underscoring that HT also carries a clear carcinogenic risk in younger populations.

Although a few prospective studies suggest that HT does not raise the rate of malignant transformation in thyroid nodules (12), the majority of clinical and pathological evidence supports that HT promotes PTC development. These findings emphasize the importance of risk assessment and surveillance for malignant transformation in HT patients with thyroid nodules.

### 2.2 Favorable prognostic impact of Hashimoto’s thyroiditis on papillary thyroid cancer

Compared to PTC without HT, patients with HT-PTC have improved outcomes, primarily reflected in more favorable clinicopathological features. A large retrospective study (n = 9, 210) demonstrated a negative association between HT and large tumor size (>4 cm), metastasis, and extrathyroidal extension. HT-PTC patients also show superior 10-year overall survival and disease-free survival. After multivariate adjustment, HT remained an independent protective factor against PTC-specific mortality (13). A multicenter retrospective study focusing on intrathyroidal PTC confirmed that HT is an independent predictor of favorable outcomes, including higher clinical remission rates and longer recurrence-free survival (14). The favorable prognostic features are well supported by multiple meta-analyses (15, 16) and may be partially attributable to the elevated TSH, TPOAb, and TgAb levels in HT (17). Beyond these immunological factors, HT-PTC patients are predominantly female and exhibit a lower frequency of *BRAF* mutations (18), suggesting that HT may synergize with sex and genetic background to improve PTC outcomes.

Although several meta-analyses have suggested a favorable prognostic role for HT, this finding is not universally consistent. One study that rigorously excluded PTC patients with high-risk pathological features found that concurrent HT was independently associated with a higher rate of distant metastasis (15.5% vs. 7.8%, p = 0.04) and shorter disease-free survival (OR = 2.17), suggesting that HT may serve as an independent predictor of recurrence and metastasis rather than a protective factor (19). Other studies have reported that the presence or absence of HT does not significantly influence PTC aggressiveness or the risk of structural disease persistence at one-year follow-up (20, 21). These retrospective studies have a lower level of evidence than meta-analyses, but they still provide important clues for understanding the origins of these discrepancies. The discrepancy appears to be driven by heterogeneity in study design, patient selection, and the definition of HT itself.

## 3 The molecular landscape of the Hashimoto’s thyroiditis-papillary thyroid cancer relationship

Current research suggests that the complex interplay between HT and PTC involves a combination of factors, including but not limited to genetic, environmental, and immunological factors.

### 3.1 Common genetic basis and driving factors

HT and PTC share overlapping genetic backgrounds, with several common genetic loci including *TSHR, BACH2, FOXE1, CLTA, EDIL3, HAPLN1*, and *HIP1*. These loci are regulated by mechanisms such as the PD-1/PD-L1 axis and methylation modifications, and have been implicated in the molecular pathways linking the two conditions (22, 23).

Regarding oncogenic drivers, *BRAF* mutations and *RET/PTC* rearrangements are well-established drivers in PTC. Both activate the RAS/MAPK pathway, thereby regulating cancer cell proliferation, differentiation, and apoptosis (24).

Gene fusions are fundamental drivers of tumorigenesis in many cancers. Jentus et al. identified an association between fusion-driven tumors and HT (25); among these fusions, *RET/PTC* rearrangements have been most extensively studied. Early research detected *RET/PTC1* and *RET/PTC3* mRNA expression in 95% of HT patients (26), indicating a possible connection between these rearrangements and the inflammatory microenvironment of HT. Subsequent clinical observations confirmed that *RET/PTC* rearrangements are more frequently detected in HT-PTC patients than in PTC alone (27). Mechanistically, evidence from *in vitro* and cell-line studies suggests that RET/PTC may promote cell proliferation and carcinogenesis through MAPK pathway activation and TERT induction (28). It has also been reported to upregulate chemokines and inflammatory mediators, potentially contributing to the establishment of a pro-inflammatory immune microenvironment within the thyroid (29, 30). This mechanism provides a molecular basis for understanding the progression of HT-associated chronic inflammation to malignancy. Notably, the clinical significance of different *RET* fusion subtypes has become a focus of recent research. Wang et al. reported that the *CCDC6-RET* fusion is more prevalent in patients with HT-PTC than with PTC alone (67.9% vs. 22.7%). In contrast, the *NCOA4-RET* fusion is associated with older age and a higher rate of lymph node metastasis. These findings imply that specific *RET* fusion subtypes may influence tumor biology in the context of HT (31).

Other receptor tyrosine kinase (RTK) fusions are increasingly recognized in association with HT-PTC. A pioneering retrospective study of pediatric PTC identified a significant correlation between RTK fusions and the coexistence of HT, extrathyroidal extension, and poorer outcomes. These alterations promote cell proliferation through MAPK pathway activation and may serve as a molecular link between the inflammatory microenvironment of HT and the aggressive behavior of PTC (32).

Regarding point mutations, *BRAF V600E* is the most common driver mutation in PTC and is typically associated with tumor aggressiveness, as confirmed by numerous studies, including meta-analyses (33, 34). Of note, the prevalence of *BRAF V600E* mutations is significantly lower in HT-PTC than in PTC alone (18, 35). This difference may partly explain the more favorable outcomes observed in HT-PTC patients. However, whether *BRAF* mutation status directly mediates the protective effect of HT remains controversial. According to a meta-analysis (36) and another clinical study (37), HT was related to less aggressive PTC regardless of *BRAF* mutation status, suggesting that its protective effect is independent of *BRAF* mutations. Thus, the precise role of *BRAF* mutations in the underlying mechanism of HT-PTC requires further investigation.

Beyond the classical driver genes discussed above, bioinformatic analyses have identified additional dysregulated genes common to both HT and PTC, potentially serving as molecular bridges between the two diseases. Among these, *SERPINA1* expression is significantly elevated in PTC tissues with an HT background and is associated with favorable outcomes, raising the possibility that it may be involved in the protective effect (38, 39). Further mechanistic studies have shown that *SERPINA1* is highly expressed in tumor-associated macrophages (TAM) (40). We therefore hypothesize that *SERPINA1* may both suppress chronic inflammation in HT and facilitate tumor immune escape, potentially promoting cancer progression. These observations highlight *SERPINA1* as a candidate molecular link between chronic inflammation in HT and the biological behavior of PTC, warranting further experimental validation.

Additionally, *LTF* and *CCL21* exhibit opposing expression patterns in HT and PTC tissues, suggesting their potential involvement in immune remodeling in this comorbidity (41). *DMBT1*, typically downregulated in thyroid cancer tissues, is elevated in HT-PTC and may improve patient outcomes by suppressing tumor progression (42). Together, these altered gene expression patterns form a molecular network linking HT and PTC, offering clues for understanding their coexistence and identifying novel biomarkers.

### 3.2 Environmental contributors: trace elements, viral infection, and lifestyle factors

#### 3.2.1 Trace elements

Imbalances in trace elements in thyroid cells drive the development of various thyroid disorders like HT and PTC. Current research indicates that iron (Fe), copper (Cu), iodine (I), cadmium (Cd), lead (Pb), selenium (Se), and other elements contribute to pathogenesis by disrupting hormone production, inactivating enzymes, and inducing cellular dysfunction (43).

Iodine is essential for thyroid hormone synthesis, participating in all steps from iodide uptake to hormone release. However, excessive iodine inhibits hormone synthesis by upregulating XBP1 expression, which suppresses the transcription of hormone synthesis-related genes, thereby offering a molecular explanation for iodine-excess thyroid dysfunction (43). Iodine also plays a critical role in the development and progression of autoimmune thyroid disorders such as HT. Epidemiological studies have linked iodine supplementation to a higher prevalence of HT (44), and animal studies suggest that exogenous iodine may enhance the immunogenicity of thyroglobulin. Iodinated thyroglobulin exposes cryptic epitopes, triggering or exacerbating thyroid autoimmune responses, which may underlie the pathogenic role of iodine in HT (45).

The relationship between iodine and PTC remains debated. Dong et al. reported an increase in PTC detection rate and HT-PTC proportion after iodine supplementation (46). However, some studies attribute this to a relative rise in PTC incidence due to a decline in other thyroid cancer subtypes (47, 48). In contrast, an *in vitro* study suggested that iodine may promote cancer cell proliferation via upregulation of the SPANXA1-PI3K/Akt pathway (49). Direct evidence is lacking on iodine’s precise role in PTC patients with HT—specifically, whether it contributes to PTC pathogenesis and prognosis by modulating autoimmune activity.

Selenium is involved in deiodinase (DIO) synthesis and redox homeostasis. Multiple studies have identified selenium deficiency as a risk factor for the development of HT (50). Further research has demonstrated that selenium modulates immune responses. In HT patients, selenium supplementation reduces TSH and autoantibody levels (51, 52), thereby alleviating hypothyroid symptoms. The mechanism may involve selenium-enhanced Treg suppressive function and downregulation of Th1/Th2-type inflammatory responses (53).

Selenium and selenoprotein levels are reduced in tumor tissues, which may be linked to tumor progression. Jonklaas et al. (54) found that serum selenium is inversely related to PTC stage, suggesting that low selenium status may promote malignant progression. Mechanistically, selenium deficiency reduces the synthesis of DIOs, leading to decreased T3 levels and compensatory elevation of TSH. This activates the cAMP signaling pathway, enhances EGF-mediated proliferation, and promotes tumor cell growth and migration. Low selenium also compromises tissue antioxidant capacity, creating a pro-carcinogenic cycle of oxidative stress driven by elevated MDA (a lipid peroxidation product) (51). In summary, selenium deficiency may contribute to HT-PTC comorbidity through a dual mechanism: promoting Th1/Th2-type inflammation and aggravating HT, while disrupting antioxidant defense barriers to create a permissive microenvironment for PTC development and progression.

**Figure 1:**
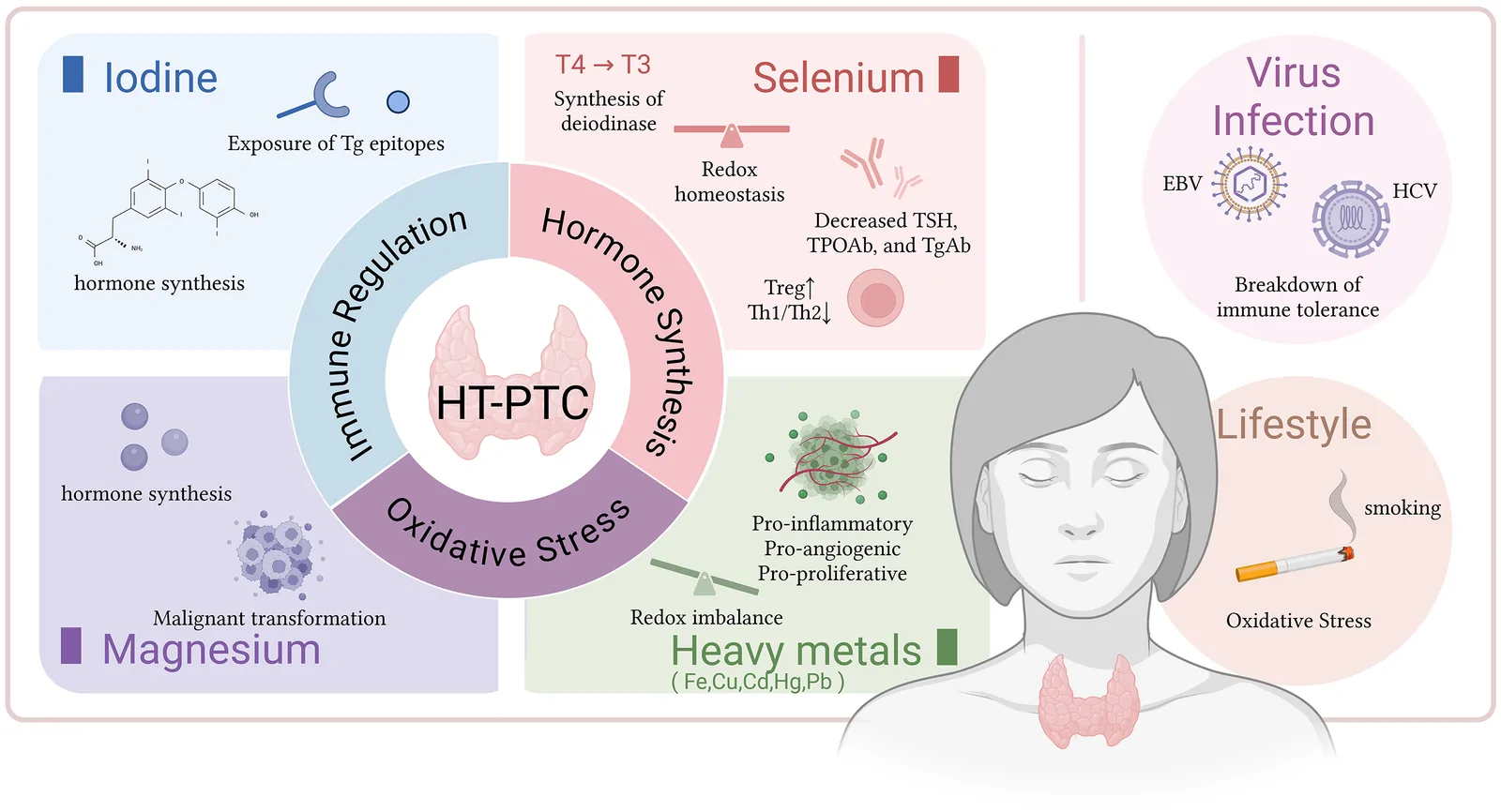
Environmental factors involved in the interplay between HT and PTC. This figure outlines an overview of environmental factors contributing to the interaction between HT and PTC, including trace elements, viral infections, and lifestyle factors. Multiple trace elements (iodine, selenium, magnesium, and heavy metals) contribute to HT and PTC pathogenesis through three primary mechanisms: immune modulation, hormone synthesis regulation, and oxidative stress mediation. Certain viruses (Epstein–Barr virus and hepatitis C virus) may disrupt immune tolerance. Unhealthy lifestyle habits, such as smoking, participate in autoimmune responses by inducing oxidative stress.

Imbalances in other trace elements also contribute to HT and PTC pathogenesis. Low magnesium is a risk factor for HT in reproductive-age women, whereas high magnesium increases the risk of thyroid nodules and malignant transformation (55, 56). Dysregulation of heavy metals such as Fe, Cu, Pb, and Cd can compromise the body’s antioxidant capacity (57). Cd, Hg, and Pb exhibit pro-inflammatory, pro-proliferative, and pro-angiogenic effects. Together, these redox imbalances and pro-tumor effects may synergistically promote PTC development and poor prognosis. However, direct evidence regarding the metabolic profiles and specific mechanisms of these trace elements in HT-PTC comorbidity remains scarce (**Figure 1**).

#### 3.2.2 Viral infection

Viral infections contribute to both autoimmune diseases and malignant tumors. Proposed mechanisms include viral latency and reactivation, which may disrupt immune tolerance and trigger autoimmune responses. Repeated cycles of inflammatory injury and repair may also increase the risk of malignant transformation. Epstein-Barr virus (EBV) has attracted substantial attention in this field. It is thought to promote cellular malignant transformation by inducing chronic inflammation and immune dysregulation (58). The high prevalence of EBV positivity and elevated EBER expression in HT-PTC tissues supports this (59, 60). Underlying mechanisms involve molecular mimicry (61), immunosuppressive cell infiltration, and pro-inflammatory effects of TAMs (58, 62). Chronic hepatitis C virus (HCV) infection is also linked to thyroid autoimmunity and tumorigenesis (63). Fallahi et al. reported that HCV patients with concurrent HT have a significantly higher PTC prevalence than controls. HCV may contribute to PTC development through CXCL10-driven Th1-type autoimmune inflammation (64). The HCV E2 protein directly activates pro-inflammatory cytokines (IL-6, IL-8, TNF-α) and autoimmune-related molecules (heat shock proteins, CD40) in thyroid cells. This inflammatory environment promotes HT and may induce genomic instability and sustained growth signals, increasing malignant transformation risk (65). Other viruses, including herpes simplex virus (HSV) and human parvovirus B19 (B19V), have also been associated with autoimmune thyroid disease and thyroid cancer (66). Overall, viruses exert multifaceted effects on the progression from HT to PTC. Current evidence, however, remains limited to small-scale data and preliminary mechanistic explorations. The OncoDB database, developed by Tang et al., integrates viral infection data with host gene expression profiles in cancer, providing a valuable platform for exploring such associations (67).

Besides, lifestyle factors such as smoking are associated with various autoimmune diseases, possibly via oxidative stress induction and autoantibody upregulation (68). However, its association with PTC risk remains debated, with several studies suggesting a potential protective effect (69).

### 3.3 Remodeling of the tumor immune microenvironment

The tumor immune microenvironment (TIME) of PTC frequently exhibits an “immune-inflamed” phenotype. Within this microenvironment, various immune cells interact with stromal cells (epithelial cells, fibroblasts, and endothelial cells) via cytokines and chemokines, driving tumor initiation and progression (70). In HT patients, extensive lymphocytic infiltration and germinal center formation create a pro-inflammatory, immune-activated microenvironment.

However, the TIME in HT is not a static entity. It evolves dynamically along both temporal and spatial axes as the disease progresses. Temporally, the immune infiltrate follows a stereotyped sequence. In early HT, macrophages and dendritic cells infiltrate the thyroid, followed by a gradual expansion of CD4^+^ T cells that drives a Th1/Th17-predominant inflammatory response (71). Over time, this diffuse lymphocytic infiltration gives way to organized lymphoid aggregates, culminating in the formation of tertiary lymphoid structures (TLS) composed of B cells and Tfh cells. This transition, from nonspecific inflammation to structured immune organization, is a key immunological milestone in HT progression. In HT-PTC, some studies have suggested a shift toward a mixed Th1/Th2 or Th2-dominant profile in patients with long-standing disease (72), although this finding has not been consistently replicated across cohorts.

At the spatial level, thyroid follicular cells (TFCs) function as central hubs that orchestrate local immune responses by recruiting and interacting with diverse immune populations. T and B lymphocytes are the principal regulators of this network, yet their contributions are functionally divergent. Among CD4^+^ T cells, Th1 and Th17 cells exert dual effects. On one hand, they drive inflammatory damage through NF-κB and PI3K/Akt signaling; on the other, they collaborate with CD8^+^ T cells and B cells to strengthen immune surveillance. Conversely, Tregs, M2-type tumor-associated macrophages (TAMs), and cancer-associated fibroblasts (CAFs) appear to favor tumor progression, an effect that may be reinforced by key checkpoints such as PD-1/PD-L1. The specific roles of these components will be elaborated in the subsequent sections.

#### 3.3.1 T cell immunity: balance among CD4^+^ T-cell subsets and the role of CD8^+^ T cells

Numerous studies reveal differences in immune cell and molecular composition between PTC with and without HT. PTC tissues with an HT background are characterized by abundant immune cell infiltration, and the density and diversity of these infiltrating immune cells correlate with favorable outcomes (73, 74).

**Figure 2:**
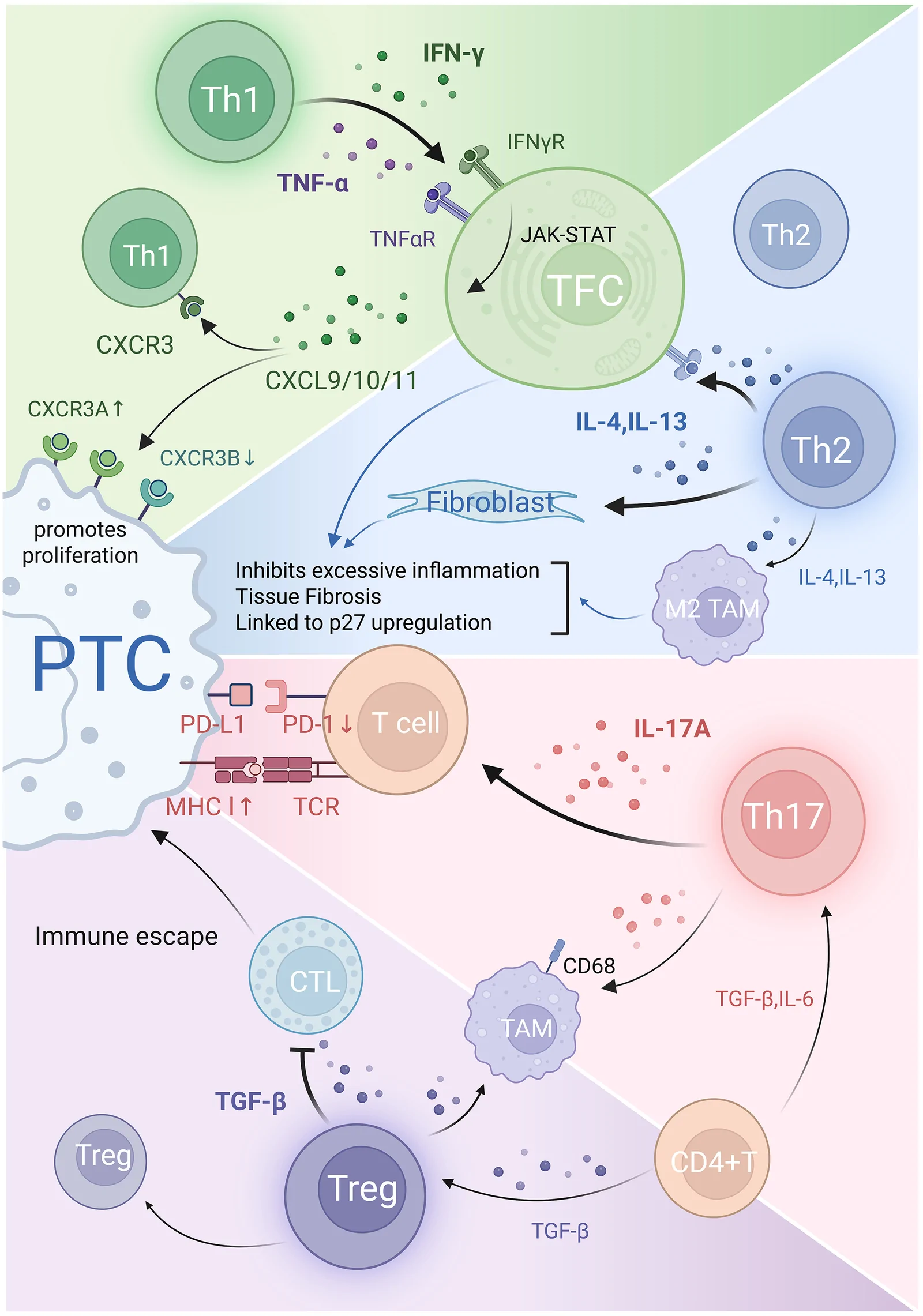
Proposed mechanisms of CD4^+^ T-cell subsets in the HT-PTC immune microenvironment. Based on currently available evidence, this schematic diagram illustrates potential mechanistic pathways through which CD4^+^ T cells may mediate the crosstalk between HT and PTC. The figure is intended to help readers understand the major hypotheses emerging from the literature; however, the pathways depicted remain largely speculative and require further experimental and clinical validation. (1) Th1: Activated Th1 cells secrete IFN-γ and TNF-α, which activate the JAK-STAT pathway in thyroid follicular cells. This induces the chemokines CXCL9, CXCL10, and CXCL11, binding to CXCR3A and CXCR3B receptors to modulate tumor immunity. (2) Th2: Th2 cells become more predominant during long-term HT-PTC coexistence. By secreting IL-4 and IL-13, they attenuate excessive inflammation, promote tissue fibrosis, and upregulate p27, which may contribute to inhibiting PTC progression. (3) Th17: Activation of Th17 cells lead to substantial IL-17A release. IL-17A downregulates PD-1 on T cells while upregulating MHC class I on PTC cells, cooperatively enhancing immunogenicity. IL-17A levels also correlate with CD68^+^ macrophage activation. (4) Treg: Treg cells often enrich locally at the tumor site in HT-PTC. By secreting TGF-β, they suppress the effector functions of CTLs and other immune cells, which may facilitate immune escape.

T cells are central to both HT pathogenesis and antitumor immune response. **Figure 2** illustrates the functions of four major CD4^+^ T cells: Th1, Th2, Th17, and Treg. In early HT, a Th1-dominant response drives chronic inflammation and thyroid destruction via pro-inflammatory cytokines such as IFN-γ and TNF-α. The chemokine axis of CXCL9/CXCL10/CXCL11 — CXCR3 establishes a positive feedback loop among Th1 cells, pro-inflammatory cytokines, chemokines, and lymphocyte recruitment, perpetuating thyroid inflammation and autoimmune damage (75, 76). Further mechanistic studies reveal that distinct CXCR3 ligands differentially program T cell functions through biased signaling. Persistently high CXCL10 expression in HT may suppress tumor growth and enhance antitumor immunity by promoting effector T cell enrichment.

Notably, CXCR3 has been reported to play a complex dual role in tumor immunity and may link Th1-type immune responses to antitumor immunity in HT. Elevated CXCR3 expression is associated with the development of various cancers, including PTC. The CXCR3-A isoform generally promotes cell proliferation and invasion, and may facilitate immune escape by recruiting suppressive cells such as Tregs. Conversely, CXCR3-B inhibits proliferation and angiogenesis while inducing apoptosis, and its frequent downregulation in cancer cells implies a tumor-suppressive potential (77–79). Thus, it is conceivable that the immune microenvironment in HT may influence PTC prognosis through more than one pathway, potentially involving CXCL10-driven immune surveillance and the CXCR3-A/B-mediated modulation of tumor behavior. However, these proposed mechanisms remain speculative and require further investigation. Moreover, the strong Th1-type immune response in HT may directly restrain tumor progression. Rotondi et al. found that IFN-γ suppresses the TNF-α/CXCL8 axis, providing a mechanistic rationale for the favorable prognosis of HT-PTC (80).

In long-standing HT with PTC, some studies have suggested that the immune microenvironment may shift from an initial Th1-dominant aggressive inflammation to a more tempered state, with concurrent Th1 and Th2 responses. HT-PTC patients exhibit significantly elevated circulating Th1 (IFN-γ), Th2 (IL-4, IL-13), and Th9-associated (IL-9) cytokines, forming a mixed but Th2-biased cytokine profile driven by upregulated STAT6 signaling (72, 81). Th2-biased inflammation may suppress PTC malignant progression through multiple mechanisms: anti-inflammatory and tissue-repair abilities may suppress Th1/Th17-mediated tissue damage; elevated TNF-α, IFN-γ, and IL-10 are associated with increased tumor suppressor p27 expression, inhibiting tumor invasion (82); and Th2-associated factors (IL-4, IL-13) may restrict local tumor invasion by promoting tissue fibrosis. These observations partially explain the favorable prognosis of HT-PTC patients. However, the mechanistic link between Th2-type immunity and HT-PTC remains incompletely understood, with insufficient causal and temporal evidence, warranting further functional and spatial multi-omics studies.

Disruption of the Th17/Treg cell balance is a central feature of immune dysregulation in HT, with multiple studies confirming a pro-inflammatory shift in the Th17/Treg ratio (83). This imbalance correlates positively with thyroid autoantibody levels and functional impairment, indicating its profound involvement in autoimmune progression (84, 85), and has also been observed in HT-PTC patients (86).

Th17 differentiation predominance in HT is driven by multiple factors. Naïve CD4^+^ T cells from HT patients are more inclined to differentiate into Th17 cells upon stimulation with thyroid autoantigens such as TPO, regulated by cytokines including IL-6 and TGF-β1 (87). Thyroid follicular cells (TFCs) can produce IL-17 in both autoimmune and cancer contexts. Elevated IL-17A expression in HT-PTC may exert a unique immunomodulatory role: it directly upregulates MHC class I expression in PTC cells to enhance immunogenicity, while promoting T cell activation and downregulating PD-1 expression (88). These findings suggest that IL-17A synergistically enhances tumor immune surveillance through dual mechanisms. Furthermore, in HT, IL-17A levels strongly correlate with the activation of CD68^+^ macrophages, suggesting that Th17 responses remodel the immune microenvironment by recruiting or activating macrophages (89).

Regulatory T cells (Tregs) and their key modulator TGF-β form a critical regulatory axis in the HT-PTC immune microenvironment, influencing the balance between autoimmunity and antitumor immunity. *In vitro* evidence shows that reducing Treg numbers promotes tumor regression but increases autoimmune susceptibility (90). The HT background reshapes Treg distribution: compared to PTC without HT, HT-PTC patients exhibit fewer peripheral Tregs but increased intrathyroidal Tregs (91, 92). This “peripheral reduction but local enrichment” pattern may reflect the compensatory Treg recruitment and expansion at sites of inflammation and tumorigenesis. While mitigating excessive autoimmune damage, this mechanism may also create a tumor-protective environment.

TGF-β is thought to serve as a key molecular hub in the above processes. In the chronic inflammatory environment of HT, TGF-β may contribute to both immune regulation and fibrosis. Although TGF-β1 is significantly upregulated in PTC tissues, its role remains complex and incompletely understood. On one hand, it has been suggested that TGF-β1 promotes Treg differentiation and functional maintenance, facilitating intratumoral Treg accumulation and immunosuppression (93). On the other hand, it may recruit immune cells, particularly CD68^+^ macrophages at the tumor margin. However, favorable prognosis correlates with intratumoral rather than margin macrophage infiltration, raising the possibility that TGF-β1 may recruit but functionally suppress macrophages, whereas macrophages that infiltrate the tumor core may represent a more cytotoxic phenotype driven by alternative signals (94). Collectively, these observations suggest that the TGF-β/Treg axis in HT-PTC may serve a dual function: suppressing autoimmunity while facilitating tumor immune escape. The balance of this axis may influence the malignant potential of PTC with HT. Of note, some studies found no significant effect of HT on Treg numbers in PTC (95), suggesting that functional status or subset composition may be more critical than quantity.

CD8^+^ cytotoxic T lymphocytes (CTLs) are markedly enriched in the TIME of HT-PTC, widely regarded as a key immunological basis for the favorable clinical outcomes of these patients (96). Histological analysis by Sulaieva et al. demonstrated increased CD8^+^ T cell infiltration in HT-PTC, with levels of tumor-aggressive suppressive cells (M2 macrophages, Tregs) relatively unchanged, suggesting HT exerts antitumor effects through cytotoxic effects (95). However, the numerical advantage of CTLs does not equal superior function. Banerjee et al. found that although CTLs were present in the TIME in 83% of cases, their activity may be suppressed by Tregs and the PD-1/PD-L1 pathway (97). Overall, the HT-PTC immune microenvironment appears to represent a state of equilibrium between immune activation and inhibition, rather than simply being ‘immune-activated’. This proposed balance may contribute to understanding the paradoxical bidirectional HT-PTC relationship: active CD8^+^ T cells may restrict rapid tumor progression, while inhibitory mechanisms prevent excessive immune attack that would either completely eliminate the tumor or trigger severe autoimmune injury.

#### 3.3.2 B cell infiltration and tertiary lymphoid structure formation

**Figure 3:**
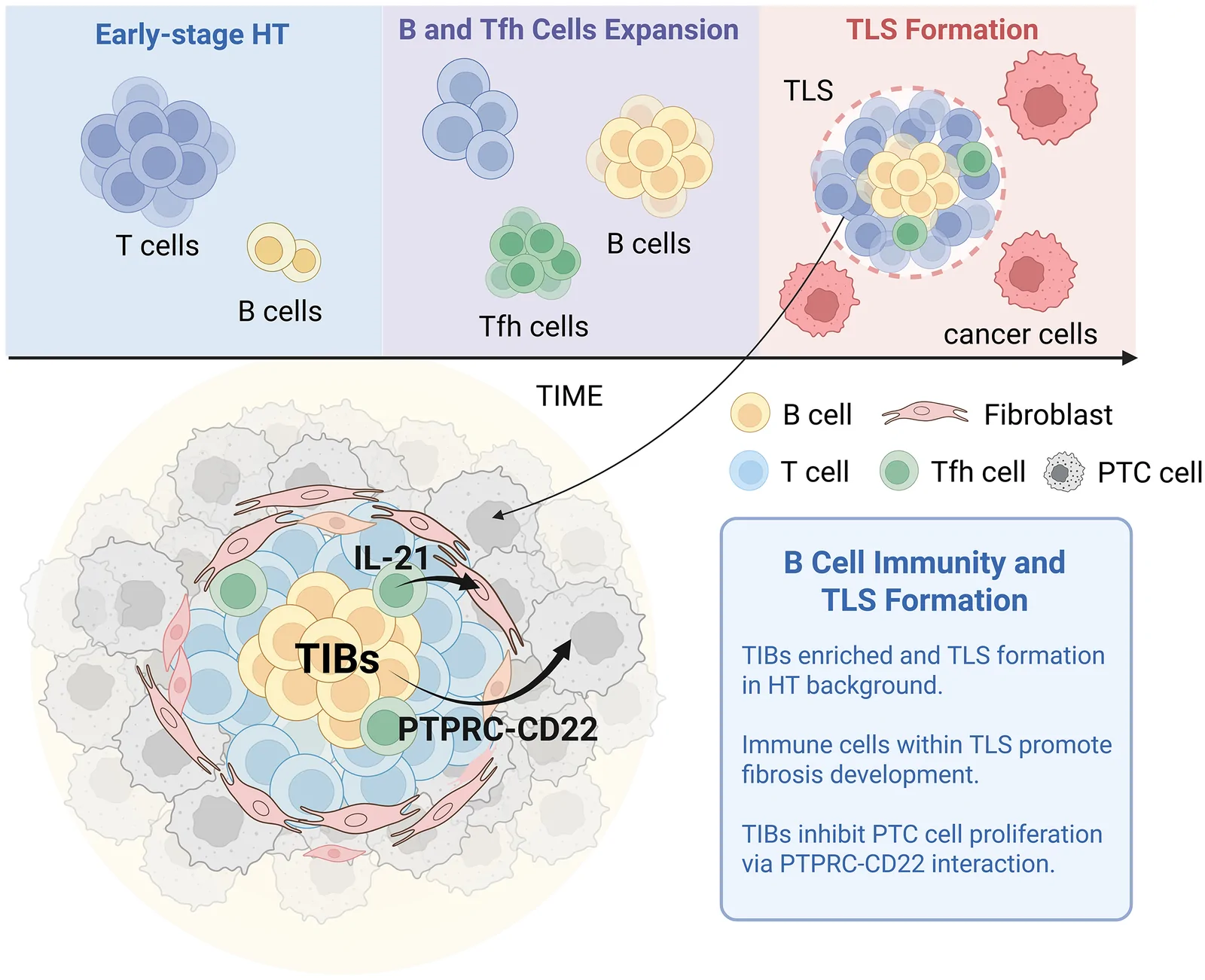
Dynamic evolution of B cells and tertiary lymphoid structures from HT to concurrent PTC. This figure illustrates the progression of B cell infiltration and tertiary lymphoid structure (TLS) formation in the thyroid from early-stage HT to HT-PTC. During HT progression, B cells and Tfh cells gradually expand and assemble with T cells and fibroblasts to form mature TLS. The figure also highlights three potential mechanisms: the activation of anti-tumor immunity, the establishment of fibrotic barriers by Tfh-derived IL-21, and the suppression of tumor cell proliferation via interactions such as the PTPRC-CD22 axis. These mechanisms, however, remain largely speculative and require further validation.

Pathological changes in AIT are characterized by pronounced B lymphocyte infiltration, which plays a central role in the disease through antigen presentation, autoantibody production, and secretion of regulatory factors. As HT progresses, the immune microenvironment may shift from T-cell-dominated to significant B-cell expansion and enrichment of follicular helper T cells (Tfh), eventually leading to the formation of tertiary lymphoid structures (TLS) within the thyroid (71, 98). TLS formation in the context of HT may profoundly influence the immune microenvironment of concurrent PTC and has been associated with favorable tumor outcomes (99). A single-cell sequencing study revealed that HT promotes the migration and infiltration of B cells and plasma cells into tumor tissues (100); These tumor-infiltrating B cells (TIBs), especially the germinal center subgroup, are significantly enriched in indolent PTC, where they form TLS and suppress tumor cell proliferation through interactions such as PTPRC-CD22 (101). Consequently, the TIME of HT-PTC is specifically enriched with TIBs and TLS, a feature rarely observed in PTC without HT (100), suggesting that HT may exert an “immune-enhancing” effect by delivering TIBs with antitumor potential to the tumor site and facilitating TLS formation, thereby establishing a suppressive immune barrier against PTC progression (**Figure 3**).

#### 3.3.3 Other immune cells and stromal cells

The interaction between immune cells and stromal cells in thyroid tissue extensively participates in the crosstalk between HT and PTC. Ma et al. identified a unique HT-associated cell population within the TIME that regulates immune-stromal interactions through an MIF-(CD74^+^CXCR4) axis-centered network (102).

Reprogramming of stromal cells, particularly fibroblasts, has been suggested to play a key role in the histopathological alterations in HT. Single-cell studies have revealed specialized stromal subsets in HT, including ACKR1^+^ endothelial cells and CCL21^+^ fibroblasts/myofibroblasts, which promote immune cell infiltration and TLS formation to facilitate the establishment of a chronic inflammatory microenvironment (103). When PTC arises in the setting of HT, this microenvironment is further remodeled by the tumor. In PTC, fibroblasts become activated and differentiate into cancer-associated fibroblasts (CAFs) that participate in extracellular matrix remodeling and angiogenesis, with several studies linking CAFs to PTC aggressiveness (104). A single-cell and spatial transcriptomic study revealed that inflammatory CAFs (iCAFs) involved in immune regulation are enriched in the HT inflammatory microenvironment. In addition, a subset of POSTN^+^ myofibroblasts (POSTN^+^ myCAFs) was tightly co-localized with invasive tumor cells and significantly associated with lymph node metastasis and poor prognosis (105). Collectively, chronic inflammation in HT may activate and remodel stromal cells, and sustained inflammatory signals during tumor progression might contribute to a pro-invasive phenotype in a subset of these cells. Notably, some studies suggest that CAFs may contribute to a suppressive TIME by forming physical barriers within TLS and recruiting inhibitory cells through paracrine signaling (106). The precise role of fibroblasts in the HT-PTC pathogenesis requires further investigation.

Tumor-associated macrophages (TAMs) are among the most abundant immune cell populations in the PTC TIME, and their polarization status profoundly influences tumor progression. TAMs are broadly classified into two major phenotypes: M1 macrophages (induced by IFN-γ, associated with pro-inflammatory responses) and M2 macrophages (induced by IL-4/IL-13, linked to anti-inflammatory and tissue-repair functions). In PTC, enrichment of M2-type TAMs is closely linked to tumor progression. In a *BRAF V600E-*driven mouse model of thyroid cancer, tumor cells highly express CSF-1 and CCL2, which recruit macrophages and induce M2 polarization; these M2-type TAMs then directly enhance tumor cell invasion by secreting factors such as CXCL8/IL-8 (107, 108). Targeting TAM polarization is a promising therapeutic strategy: an animal study demonstrated that lactoferrin immune complexes (LTF) can reprogram M2-type TAMs toward an M1-like phenotype to inhibit tumor growth (109), while breast cancer models showed that inhibiting STAT6 (a key driver of M2 polarization) blocks M2 macrophage differentiation and tumor growth/metastasis. These results indicate a clear therapeutic direction for thyroid tumors with a comparable TAM polarization shift (110).

Other innate immune and myeloid cells also form complex regulatory networks in the HT-PTC microenvironment. Functional impairment of dendritic cells often contributes to tumor immune escape (111). Mast cell infiltration is increased in PTC, and under HT-related chronic inflammation, mast cells may enhance tumor malignancy by promoting proliferation, angiogenesis, and epithelial-mesenchymal transition (EMT) (108, 112). Myeloid-derived suppressor cells accumulate in chronic inflammatory conditions such as HT and suppress immune responses through multiple mechanisms, including the PD-1/PD-L1 pathway, establishing a broadly immunosuppressive environment that favors PTC development (113). Together, these three cell types contribute to an immunosuppressive state within the autoimmune milieu that supports tumor survival.

#### 3.3.4 Key immune checkpoint: the PD-1/PD-L1 pathway

Within the complex interplay between HT and PTC, the PD-1/PD-L1 immune checkpoint acts as a tightly regulated and functionally multifaceted node. Its expression and function display a distinct pattern in the HT background, profoundly influencing the balance of the TIME. HT is an important microenvironmental factor of PD-L1 expression in PTC. Research has demonstrated that sustained high expression of PD-L1 by tumor cells is more frequently observed when PTC arises in the setting of HT, and is correlated with metastatic potential, whereas PTC in normal thyroid or other types of thyroiditis generally does not express PD-L1 (114). Further analysis identified HT with eosinophilic metaplasia as an independent predictor of PD-L1 expression in PTC (115), providing evidence for HT involvement in tumor immunity through the PD-1/PD-L1 pathway.

Notably, the function of the PD-1/PD-L1 axis in HT-PTC varies by cell type and associated upstream/downstream signals. In Th1-type inflammation, abundant IFN-γ in HT induces PD-L1 expression on TFCs, including cancer cells, as a mechanism to restrict excessive intrathyroidal autoimmunity. However, cancer cells may bind PD-L1 to PD-1 on infiltrating T cells to actively suppress antitumor immune response and achieve immune escape (116, 117). In Th17 and Treg cells, PD-1 expression levels are altered: Th17 cells in HT exhibit low PD-1 expression, which may contribute to excessive inflammatory responses, whereas Tregs — despite their lower percentage in HT — show abnormally high PD-1/PD-L1 levels. The paradoxical finding suggests a dysfunctional or inadequately compensatory pathway in Tregs, weakening their immunosuppressive capacity (83). Together, these disturbances exacerbate the breakdown of immune homeostasis.

Additionally, the canonical driver mutation *BRAF V600E* in PTC induces PD-L1 expression through MAPK pathway activation (118), suggesting that in HT-PTC, chronic inflammatory signals (such as IFN-γ) and oncogenic signaling may synergistically reinforce PD-1/PD-L1 axis-mediated immune escape.

#### 3.3.5 Signaling pathways: NF-κB and PI3K/Akt

NF-κB and PI3K/Akt are two core signaling pathways linking HT-related chronic inflammation to PTC development and progression. As a well-established inflammatory switch, NF-κB is abnormally activated in HT, driving pro-inflammatory cytokine expression and aggravating autoimmune damage. In HT-PTC, tumor cells may specifically activate the NF-κB pathway through oncogenic drivers (e.g., *RET/PTC, BRAF*) and mechanisms such as CD25 (IL-2 receptor) expression, sustaining a pro-inflammatory microenvironment that favors tumor cell survival and proliferation (119, 120). The PI3K/Akt pathway represents another molecular bridge in HT-PTC: a study has shown that phosphorylated Akt and its isoforms (Akt1, Akt2) are significantly upregulated in both HT and PTC tissues, providing potent survival and proliferative signals to HT-PTC (121).

### 3.4 Thyroid function, thyroid-stimulating hormone, and autoantibodies

The risk of thyroid cancer in patients with HT is closely related to preserved thyroid function. Paparodis et al. found that cancer risk was significantly increased only in euthyroid or mild hypothyroid individuals, but not in those with complete loss of function or high TPOAb levels (122).

Elevated TSH, a hallmark of hypothyroidism resulting from HT, is consistently recognized as an independent predictor of PTC (123, 124). As a potent growth factor for TFCs, TSH promotes thyroid cancer initiation and progression by activating signaling pathways such as cAMP/PKA and MAPK through its receptor (TSHR) (125). An animal study further revealed that TSH drives tumorigenesis by disrupting the p53-dependent senescence barrier induced by oncogenes such as *BRAF V600E*, allowing proliferation of cells carrying driver mutations (126). The impact of TSH on PTC aggressiveness remains unclear (127), possibly due to a counteracting interplay between TSHR-mediated pro-proliferative effects and HT-associated immune responses.

The relationship between thyroid autoantibodies and PTC development and progression is complex. While high TPOAb levels serve as a protective factor against PTC development in HT patients (122) and correlate negatively with extrathyroidal extension and lymph node metastasis (128), it is also associated with multifocality and an increased risk of cancer recurrence (129–131). TgAb positivity is associated with PTC development and poor prognosis; therefore, postoperative TgAb positivity can predict persistent or recurrent disease (128, 132, 133).

Emerging evidence challenges a simplistic binary view of autoantibodies as either risk or protective factors, suggesting that distinct autoantibody profiles may correspond to different immune microenvironments. Wen et al. found that both double-negative and double-positive status for TPOAb and TgAb were associated with a high risk of central lymph node metastasis (CLNM) in HT-PTC (134). Conversely, Lu et al. reported that double-negative status was associated with large tumor volume and lymph node metastasis, whereas double-positive status and isolated TPOAb positivity exhibited a protective association (135). These findings highlight the importance of integrating preoperative antibody profiling into a personalized risk assessment system for HT-PTC.

### 3.5 Noncoding RNA regulatory networks

Non-coding RNAs (ncRNAs), including microRNAs (miRNAs) and long non-coding RNAs (lncRNAs), have provided new insights into the complex interplay between HT and PTC. These regulatory molecules are often carried by exosomes and play important roles in intercellular communication. Dysregulated MiRNAs are observed in both HT and PTC, with the miR-200 family becoming increasingly recognized. *In vitro* evidence suggests that miR-200b suppresses EMT in primary PTC lesions (136), exerting a tumor-suppressive effect. However, in HT-PTC, this activity may be counteracted by ADAR1, which is upregulated in response to HT-related inflammatory signaling (e.g., RAS-PI3K activation). ADAR1 is thought to inactivate miR-200b through A-to-I editing, thereby potentially facilitating EMT and tumor progression (137). This provides a novel mechanism underlying HT-PTC aggressiveness and positions ADAR1 as a potential therapeutic target. However, this proposed mechanism is largely derived from cell-line experiments and awaits further validation in patient tissues and *in vivo* models.

Competing endogenous RNA (ceRNA) networks comprising mRNAs, miRNAs and lncRNAs are key components of the EMT regulatory circuitry and involved in tumorigenesis (138). Expression levels of multiple miRNAs and lncRNAs differ significantly between PTC patients with and without HT. For instance, lncRNA ENST00000452578 is downregulated in HT-PTC and negatively correlates with tumor size and proliferation, suggesting a potential protective role (139); another lncRNA, *BRAF*-activated non-coding RNA (BANCR), is implicated in various cancers and may influence surface signaling such as MAPK in HT-PTC (140, 141). Additionally, lncRNAs can specifically regulate chemokine and chemokine receptor mRNAs (e.g., CXCL10, CXCL9, and CCR2) in the TIME by ceRNA mechanisms, thereby recruiting/activating Th1-type immune cells and remodeling the immune-inflammatory microenvironment (142). However, studies in this area remain limited in number, and the overall level of evidence is low, warranting cautious interpretation. Given the ability of ncRNAs to regulate gene expression, targeting specific ncRNAs has become a potential therapeutic strategy for both HT and PTC, although clinical application remains limited by delivery efficiency and immune rejection (143).

### 3.6 Gut microbiota and the gut-thyroid axis

The gut-thyroid axis is an emerging area of research, and the gut microbiota has been implicated in the pathogenesis of both autoimmune diseases and cancer (144). Mendelian randomization studies have suggested potential associations between specific microbiota, such as Desulfovibrionaceae and Prevotella_7, and the risk of thyroid nodules and cancer (145, 146).

HT patients exhibit altered gut microbiota diversity and composition. Dysbiosis, overgrowth of harmful microbes, impaired trace element absorption, and changes in intestinal permeability collectively contribute to HT development (147–149). Certain genera such as Bacteroides and Lactobacillus have been associated with TSH and autoantibody levels (150). The gut microbiota also influences the immune microenvironment in HT (149). Wu et al. reported that early pregnancy hypothyroidism is associated with gut dysbiosis (increased Prevotella and Faecalibacterium, decreased Bacteroides), which significantly correlated with a shift toward Th1-dominance in the peripheral Th1/Th2 immune balance (151). Dysregulation of the gut microbiota and its metabolites—particularly short-chain fatty acids—has been reported to modulate the Th17/Treg immune balance and systemic chronic inflammation, potentially contributing to cancer development through mechanisms such as intestinal barrier disruption and oxidative stress (152, 153).

A metagenomic and transcriptomic study revealed significant interactions between key gut microbiota and the host transcriptome (mRNAs and miRNAs) in early-stage HT, regulating pathways involved in immunity and cancer (148). Dysbiosis may influence cancer development and progression through interactions with ncRNA expression and related signaling pathways, although direct evidence in HT-PTC is currently lacking (154). This hypothetical link connecting autoimmunity, gut microbiota, ncRNAs, and cancer offers a new perspective for understanding the mechanisms in HT-PTC comorbidity.

### 3.7 Additional mechanisms: inflammasomes, epithelial-mesenchymal transition, oxidative stress and fetal microchimerism

In addition to well-established genetic and immunological factors, emerging research is offering fresh perspectives on the complex molecular mechanisms linking HT and PTC.

#### 3.7.1 Inflammasomes and pyroptosis

In HT pathogenesis, inflammasome pathways (e.g., NLRP3, AIM) in TFCs are abnormally activated, accompanied by characteristic pyroptosis, and activation correlates with autoantibody levels (155). Sustained activation of this process is positively regulated by the deubiquitinating enzyme USP1, which amplifies inflammatory signals by stabilizing inflammasome proteins and promoting their transcription (156). Emerging evidence from human thyroid tissue studies suggests that this USP1-associated inflammasome-pyroptosis axis may exert a dual influence on the TIME. This pathway may promote PTC development through chronic inflammation and tissue regeneration, while potentially enhancing antitumor immunity through antigen release from dying cells. It has therefore been suggested that this axis could act as a potential switch influencing whether the HT microenvironment shifts toward a pro- or anti-tumorigenic state. However, whether its dysregulation directly contributes to PTC development or affects prognosis in HT patients remains to be determined (157). Novel strategies targeting this axis—such as USP1 or pyroptosis inhibition—offer a novel strategic direction for HT intervention and PTC risk management.

#### 3.7.2 Epithelial-mesenchymal transition

HT may alter the inner properties of tumor cells by suppressing EMT, potentially providing a cellular basis for the favorable prognosis observed in HT-PTC. During early progression from HT to PTC, chronic inflammation may upregulate the adhesion molecule ICAM-1 and contribute to initial local invasion (158). However, in coexisting HT-PTC, the molecular profile of tumor cells undergoes significant remodeling: mesenchymal markers ICAM-1 and N-cadherin are downregulated, while the epithelial marker E-cadherin is markedly increased; suppressed EMT and reduced migratory capacity in these tumor cells are potentially driven by downstream alterations in signaling pathways such as MAPK/Erk and PI3K/Akt (159, 160). This altered molecular profile has been linked to the differentially expressed gene ANK3 in HT-PTC (161). These dynamic changes in EMT may partly explain the dual role of HT in PTC.

#### 3.7.3 Oxidative stress

In patients with HT-PTC, genes related to oxidative stress and reactive oxygen species (ROS) are significantly differentially expressed, suggesting their involvement in linking these two conditions (162). Lopes et al. found that PTC patients without HT exhibited higher oxidative stress and angiogenic activity than those with concurrent HT, suggesting that HT may reduce oxidative stress and its downstream effects in PTC (163). Peroxiredoxin 2 (PRDX2) exerts antitumor effects by modulating oxidative stress and inflammatory signaling, positioning it as a shared protective factor in both HT and PTC (164). The autoimmune environment in HT may thus suppress the pro-tumorigenic effects of oxidative stress and reduce PTC aggressiveness.

In addition, an early study reported enrichment of fetal microchimeric cells in thyroid tissues of patients with AITD and thyroid cancer, raising the possibility of their involvement in autoimmunity and tumor modulation (165). Nevertheless, the lack of recent mechanistic studies leaves this hypothesis largely unexplored.

## 4 Discussion

### 4.1 Summary and innovations

**Figure 4:**
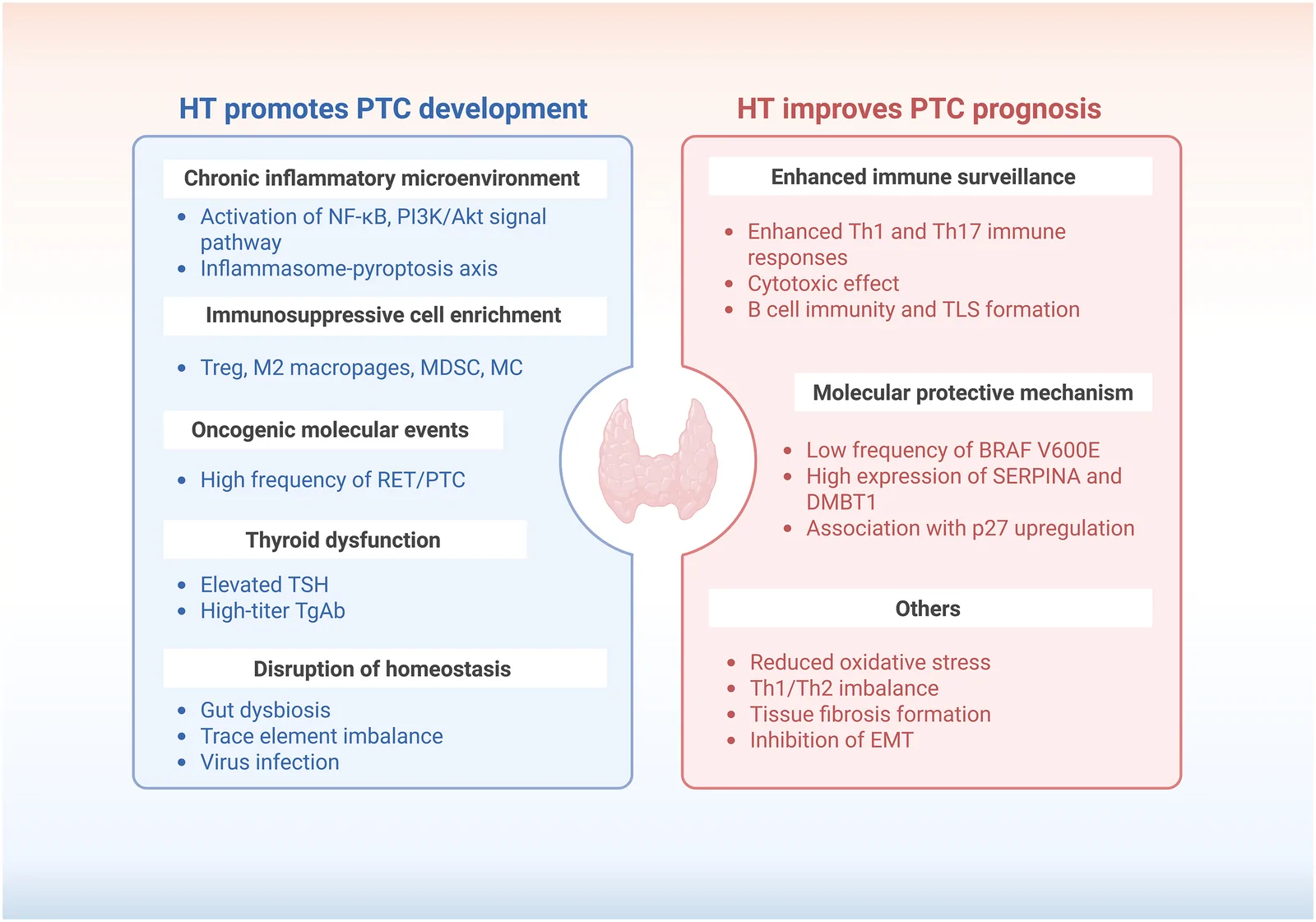
The dual role of HT in PTC: from pathogenesis to prognosis. This figure summarizes the potential factors through which HT may both promote PTC development and contribute to a more favorable prognosis. Mechanisms underlying HT-mediated PTC progression include chronic inflammation (NF-κB, inflammasome-pyroptosis), immunosuppressive cells (Tregs, M2 macrophages), oncogenic factors (RET/PTC), elevated TSH/TgAb, and environmental/microbial disturbances. Conversely, the favorable prognosis in HT-PTC may be attributed to enhanced immune surveillance (proinflammatory T cells, B cells, TLS), protective molecular signatures (BRAF V600E, SERPINA, DMBT1), and other mechanisms (reduced oxidative stress and EMT).

The HT-PTC association is best understood as a dual-edged phenomenon shaped by bidirectional remodeling of the TIME. On one side, Th1, Th17, and TIBs establish a surveillance network that constrains tumor progression. On the other, chronic inflammation, PD-1/PD-L1 activation, and suppressive cells create a permissive milieu for tumor development. These immunological forces are further modulated by genetic, environmental, epigenetic, thyroid, and microbial factors (**Figure 4**). A structured summary of the main findings is provided in **Supplementary Table 1**.

Building on previous work, this review systematically synthesizes recent literature addressing the paradoxical “double-edged” relationship between HT and PTC. We not only compile conflicting evidence and evaluate its reliability, but also dissect the sources of heterogeneity, emphasizing the need for careful patient stratification in future studies of HT-PTC comorbidity. Second, for the first time, we delineate the spatiotemporal evolution of the TIME across early-stage HT, late-stage HT, and HT-PTC coexistence, and propose a hierarchical network model with TFCs as central effectors and multiple immune cell populations as key regulators. This framework helps fill a gap in the integrated understanding of TIME in HT-PTC. Furthermore, we incorporate gut microbiota, ceRNA networks, and trace elements into the regulatory landscape, establishing a comprehensive framework that spans clinical, genetic, immunological, environmental, and microbial dimensions.

### 4.2 The paradoxical effect of HT on PTC prognosis and sources of heterogeneity

Although meta-analyses consistently suggest that HT is associated with a favorable prognosis in PTC, this conclusion is not universally supported. Several single-center studies have reported conflicting results, and the origins of these discrepancies are likely rooted in heterogeneity across study populations—particularly in age, autoantibody profiles, and tumor subtypes.

Age appears to be a major determinant of this variability. In patients under 55 years, HT has been identified as a strong risk factor for aggressive tumor behavior, whereas no such association was observed in older patients (166, 167). This age-dependent effect may be explained, at least in part, by immunosenescence: HT patients exhibit an exhausted T-cell phenotype, and functionally impaired T cells may attenuate pro-tumorigenic inflammation while tolerating autoimmune responses (168). Beyond age, autoantibody profiles add another layer of complexity. The combined status of TgAb and TPOAb, rather than either antibody alone, correlates with metastatic risk, with both double-negative and double-positive statuses associated with a higher risk of central lymph node metastasis (134). These profiles may represent two extremes—loss of immune surveillance versus excessive autoimmune activation—either of which could favor tumor progression.

Small-scale clinical studies have also suggested that obesity (169) and the IgG4-positive HT subtype (170) may be linked to worse tumor outcomes, possibly through an additive effect of chronic inflammation and HT-related autoimmunity. The association between HT and multifocality or central lymph node metastasis was observed only in the papillary thyroid microcarcinoma (PTMC) subgroup, but not in conventional PTC (171). This finding may seem counterintuitive. One possible explanation is that PTMC, though typically indolent, can be driven to metastasize by HT-related inflammation, thereby revealing its otherwise indolent behavior. However, these interpretations remain hypothesis-generating and require validation in large-scale cohort studies and mechanistic experiments.

### 4.3 Strengths and limitations of the current evidence

The evidence base for the HT-PTC association is heterogeneous, spanning from well-established clinical observations to hypothesis-generating mechanistic proposals. At the highest level of certainty, multiple clinical studies and meta-analyses have consistently shown that HT increases the risk of PTC and is associated with a favorable prognosis and a lower prevalence of *BRAF V600E* mutations. These findings represent the most reliable evidence currently available and provide the clinical foundation for the dual-effect model of HT in PTC.

At the intermediate level, evidence concerning *RET* rearrangements, immune microenvironment remodeling, TSH, and thyroid autoantibodies is largely derived from small single-center studies, *in vitro* experiments, or animal models. Although these studies have generated plausible mechanistic hypotheses, such as the Th1-CXCL9/10/11-CXCR3 axis and the inflammasome–pyroptosis pathway, their clinical relevance remains to be established. At the lowest level of certainty are findings from bioinformatic analyses and indirect observations, including the proposed roles of *SERPINA1*, *DMBT1*, the TGF-β/Treg axis, gut microbiota, and trace elements. These hypotheses offer intriguing new perspectives but remain largely speculative and require functional validation in human tissues and experimental models. To facilitate direct comparison of the available evidence on these controversial topics, we have summarized the study design, population, sample size, main findings, direction of effect, and evidence level in **Supplementary Table 2**.

Several limitations of the current literature deserve consideration. Most studies focus on clinical association, lacking temporal evidence for causality and valid animal models replicating disease progression. HT heterogeneity is often neglected; failure to stratify patients by factors such as autoantibody profiles, thyroid function status, and immune subtypes may explain conflicting findings in the literature. Furthermore, how HT remodels the TIME remains unclear, with current knowledge limited to bulk tissue analysis. Addressing these gaps will require future studies that integrate well-designed translational and interventional approaches to validate the mechanistic hypotheses discussed in this review.

### 4.4 Clinical implications and future directions

Clinically, HT poses several challenges in PTC management. It reduces the accuracy of thyroid ultrasound, leading to a higher false-positive rate and lower specificity (172), thus compromising the reliability of the TI-RADS scoring system. HT may also influence the efficacy of radioactive iodine therapy for PTC, although evidence remains inconclusive (168, 173). Besides, HT interferes with molecular testing: it reduces the sensitivity of *BRAF V600E* mutation detection in HT-PTC, whereas high-frequency mutational targets, such as *RET* rearrangements, may serve as complementary molecular markers.

Multimodal ultrasound and artificial intelligence may offer partial solutions to HT-PTC diagnostic challenges. DuanYang et al. found that elastography combined with contrast-enhanced ultrasound improves diagnostic performance (174), and AI algorithms trained on data from various HT subtypes may enhance the differentiation between benign and malignant lesions (175). Nevertheless, establishing HT-specific diagnostic criteria through multidisciplinary collaboration remains essential. Future work should prioritize HT-PTC-specific molecular tests, risk assessment incorporating HT features, and therapies targeting the tumor microenvironment, to facilitate personalized diagnosis and treatment for HT-PTC patients.
