# Supplementary material for: Hashimoto’s thyroiditis both promotes and protects against papillary thyroid cancer
Source: Front Endocrinol (Lausanne). 2026 Sep 2;17:1898677. doi: 10.3389/fendo.2026.1898677 (PMC13579237; doi:10.3389/fendo.2026.1898677)
Supplement: Supplementary file 1 [file Table1.docx]

**Supplementary Table S1. Summary of Key Findings on the HT-PTC Association**

| **Topic** | **Key Finding** | **Direction of Effect** |
| --- | --- | --- |
| **Clinical Features** |  |  |
| PTC multifocality/bilaterality | Higher rates of bilateral/multifocal PTC in HT | Risk-increasing |
| PTC prognosis | Meta-analyses: better prognosis with HT (lower ETE, LN metastasis, recurrence); conflicting reports exist | Controversial |
| **Genetic Features** |  |  |
| *BRAF V600E* mutation | Lower mutation rate in HT-PTC; protective effect independent of BRAF status | Neutral |
| *RET/PTC* rearrangements | More frequent in HT-PTC; fusion subtypes may differentially affect tumor behavior | Risk-increasing |
| SERPINA1 | Upregulated in HT-PTC; linked to both tumor development and better outcomes | Controversial |
| **Immune Microenvironment** |  |  |
| Th1-CXCR3 axis | Mediates both inflammatory damage and antitumor immunity | Controversial |
| Th2/IL-4/IL-13 | Late-stage HT; suppress invasion via fibrosis and p27 upregulation | Protective |
| Th17/IL-17A | Enhances immune surveillance by upregulating MHC I and downregulating PD-1 | Protective |
| Treg/TGF-β | Locally enriched in HT-PTC; suppress CTLs via TGF-β | Risk-increasing |
| CD8⁺ CTLs | Enriched in HT-PTC with functional cytotoxic activity | Protective |
| B cells/TLS | TIBs and TLS enriched in HT-PTC; suppress tumor proliferation | Protective |
| M2-type TAMs | Promote invasion and immune suppression via CXCL8/IL-8 | Risk-increasing |
| CAFs | POSTN⁺ myCAFs associated with invasiveness and LN metastasis | Risk-increasing |
| PD-1/PD-L1 axis | HT-driven PD-L1 expression; synergistically activated by IFN-γ and BRAF V600E | Risk-increasing |
| **Environmental & Hormonal Factors** |  |  |
| Elevated TSH | Drives tumorigenesis via TSHR-cAMP/PKA pathway | Risk-increasing |
| Thyroid autoantibodies | TgAb+ linked to poor prognosis; TPOAb role controversial; complex effects with antibody combinations | Risk-increasing |
| Selenium | Deficiency promotes HT; supplementation reduces TSH and autoantibodies | Protective |
| Iodine | Excess may trigger autoimmunity via cryptic Tg epitopes | Risk-increasing |
| Heavy metals | Cd, Pb, Hg show pro-inflammatory/pro-proliferative effects | Risk-increasing |
| **Non-coding RNAs** |  |  |
| ADAR1/miR-200b axis | ADAR1 upregulation inactivates miR-200b, promoting EMT | Risk-increasing |
| ceRNA network (lncRNAs) | Low lncRNA expression associated with reduced invasiveness | Protective |
| **Gut Microbiota** |  |  |
| Gut dysbiosis | Altered microbiota composition may regulate immune/cancer pathways | Controversial |
| **Additional Mechanisms** |  |  |
| Inflammasome-pyroptosis axis | USP1 drives aberrant inflammasome activation; may be a key switch for HT-to-PTC transformation | Controversial |
| EMT suppression | HT inhibits EMT (↑ E-cadherin, ↓ mesenchymal markers), contributing to better prognosis | Protective |

Direction of effect indicates the association between the factor and PTC risk or prognosis. "Protective" refers to an association with reduced risk or improved prognosis; "risk-increasing" refers to an association with increased risk or worse prognosis; "neutral" indicates no significant association; "controversial" indicates conflicting or bidirectional findings across studies. HT, Hashimoto's thyroiditis; PTC, papillary thyroid cancer; ETE, extrathyroidal extension; LN, lymph node; TIBs, tumor-infiltrating B cells; TLS, tertiary lymphoid structures; TAMs, tumor-associated macrophages; CAFs, cancer-associated fibroblasts; EMT, epithelial–mesenchymal transition; TgAb, thyroglobulin antibody; TPOAb, thyroid peroxidase antibody.

**Supplementary Table S2. Evidence Summary for Controversial Topics in the HT-PTC Association.**

| **Topic** | **Study Design** | **Population** | **Sample Size** | **Main Findings** | **Direction** | **Classification** | **Level** |
| --- | --- | --- | --- | --- | --- | --- | --- |
| **Prognosis** | Meta-analysis (Moon, 2018) | HT-PTC vs PTC | 11,132 vs 32,902 | ↓ ETE, ↓ LN metastasis, ↓ distant metastasis, ↓ recurrence | Protective | Clinical | High |
|  | Meta-analysis (Lee, 2013) | HT-PTC vs PTC | 2,471 vs 8,177 | ↓ ETE, ↓ LN metastasis, ↑ RFS | Protective | Clinical | High |
|  | Retrospective (Marongiu, 2023) | HT-PTC (low-risk excluded) | 97 vs 206 | ↑ metastasis (OR=2.17), ↓ DFS | Risk-increasing | Clinical | Moderate |
|  | Retrospective (Nasiri, 2023) | HT-PTC vs PTC | 50 vs 39 | No significant differences | Neutral | Clinical | Moderate |
| **BRAF V600E** | Meta-analysis (Kim, 2016) | HT-PTC vs conv. PTC | Total 3,332 | Lower BRAF V600E in HT-PTC; protective effect independent of BRAF status | Neutral | Clinical | High |
| **SERPINA1** | Bioinformatics (Liu et al., 2023 et al.) | HT-PTC vs PTC | Multiple cohorts | Upregulated in HT-PTC; linked to both development and better prognosis | Controversial | Speculative | Low |
| **Iodine** | Retrospective (Dong, 2013) | PTC pre/post iodine | Total 1,239 | ↑ HT-PTC proportion after iodine supplementation | Protective | Clinical | Moderate |
|  | In vitro (Yang, 2019) | Cell lines | — | Promotes proliferation via SPANXA1-PI3K/Akt | Risk-increasing | Experimental | Low |
| **Th1-CXCR3 axis** | Clinical (Urra et al., 2017) | Thyroid neoplasia | 123 | CXCR3-A promotes proliferation; CXCR3-B suppresses tumor growth | Controversial | Clinical + Experimental | Moderate |
| **CD8⁺ CTLs** | Clinical (Sulaieva, 2020; Banerjee, 2022) | HT-PTC vs PTC | Multiple cohorts | Enriched in HT-PTC with low exhaustion markers; may be suppressed by Tregs/PD-1 | Protective | Clinical | Moderate |
| **TIBs/TLS** | Single-cell (Pan, 2021; Li, 2025) | HT-PTC tissues | Multiple cohorts | TIBs/TLS enriched in HT-PTC; suppress proliferation via PTPRC-CD22 | Protective | Experimental | Moderate |
| **PD-1/PD-L1** | Clinical (Lubin, 2018; Alvarez-Sierra, 2019) | HT-PTC tissues | Multiple cohorts | HT drives PD-L1; IFN-γ and BRAF V600E synergistically activate axis | Risk-increasing | Clinical + Experimental | Moderate |
| **Autoantibodies** | Case-control (Tan, 2024) | PTC patients | Total 15,305 | High TgAb → aggressive features; high TPOAb → less aggressive | Controversial | Clinical | Moderate |
|  | Meta-analysis (Lee, 2020) | Thyroid cancer | Not specified | TgAb+ and persistent/increasing trends → poor prognosis | Risk-increasing | Clinical | High |
|  | Retrospective (Wang, 2022) | PTC patients | Total 5,770 | TPOAb+ → postoperative recurrence risk | Risk-increasing | Clinical | Moderate |
|  | Retrospective (Wen, 2019; Lu, 2025) | HT-PTC patients | 346 + 317 | Complex associations with different antibody combinations | Controversial | Clinical | Moderate |
| **ncRNAs (ADAR1)** | In vitro (Ramírez-Moya, 2020) | PTC cell lines | — | ADAR1/miR-200b → EMT and tumor progression | Risk-increasing | Experimental | Low |
| **ncRNAs (ceRNA)** | In vitro & bioinformatics (Zhang, 2022) | HT-PTC tissues | Multiple cohorts | Low lncRNA expression → reduced invasiveness | Protective | Experimental + Speculative | Low |
| **Gut microbiota** | Multi-omics (Li, 2024) | HT vs healthy | Large-scale | Gut dysbiosis interacts with mRNA/miRNA; regulates immune/cancer pathways | Controversial | Speculative | Low |

Direction of effect indicates the association between the factor and PTC risk or prognosis. "Protective" refers to an association with reduced risk or improved prognosis; "risk-increasing" refers to an association with increased risk or worse prognosis; "neutral" indicates no significant association; "controversial" indicates conflicting or bidirectional findings across studies. Evidence classification: Clinical = derived from human observational or interventional studies; Experimental = derived from in vitro, animal, or single-cell studies; Speculative = derived from bioinformatics or indirect evidence. Level of evidence reflects the authors' assessment of the strength and consistency of the available evidence. HT, Hashimoto's thyroiditis; PTC, papillary thyroid cancer; ETE, extrathyroidal extension; LN, lymph node; DFS, disease-free survival; RFS, recurrence-free survival; TIBs, tumor-infiltrating B cells; TLS, tertiary lymphoid structures; TgAb, thyroglobulin antibody; TPOAb, thyroid peroxidase antibody; EMT, epithelial–mesenchymal transition.
